# Molecular Characterization and Analysis of a Novel Protein Disulfide Isomerase-Like Protein of *Eimeria tenella*

**DOI:** 10.1371/journal.pone.0099914

**Published:** 2014-06-16

**Authors:** Hongyu Han, Hui Dong, Shunhai Zhu, Qiping Zhao, Lianlian Jiang, Yange Wang, Liujia Li, Youlin Wu, Bing Huang

**Affiliations:** Shanghai Veterinary Research Institute, Chinese Academy of Agricultural Sciences, Key Laboratory of Animal Parasitology of Ministry of Agriculture, Minhang, Shanghai, PR China; Instituto de Biociencias - Universidade de São Paulo, Brazil

## Abstract

Protein disulfide isomerase (PDI) and PDI-like proteins are members of the thioredoxin superfamily. They contain thioredoxin-like domains and catalyze the physiological oxidation, reduction and isomerization of protein disulfide bonds, which are involved in cell function and development in prokaryotes and eukaryotes. In this study, EtPDIL, a novel PDI-like gene of *Eimeria tenella*, was cloned using rapid amplification of cDNA ends (RACE) according to the expressed sequence tag (EST). The EtPDIL cDNA contained 1129 nucleotides encoding 216 amino acids. The deduced EtPDIL protein belonged to thioredoxin-like superfamily and had a single predicted thioredoxin domain with a non-classical thioredoxin-like motif (SXXC). BLAST analysis showed that the EtPDIL protein was 55–59% identical to PDI-like proteins of other apicomplexan parasites. The transcript and protein levels of EtPDIL at different development stages were investigated by real-time quantitative PCR and western blot. The messenger RNA and protein levels of EtPDIL were higher in sporulated oocysts than in unsporulated oocysts, sporozoites or merozoites. Protein expression was barely detectable in unsporulated oocysts. Western blots showed that rabbit antiserum against recombinant EtPDIL recognized only a native 24 kDa protein from parasites. Immunolocalization with EtPDIL antibody showed that EtPDIL had a disperse distribution in the cytoplasm of whole sporozoites and merozoites. After sporozoites were incubated in complete medium, EtPDIL protein concentrated at the anterior of the sporozoites and appeared on the surface of parasites. Specific staining was more intense and mainly located on the parasite surface after merozoites released from mature schizonts invaded DF-1 cells. After development of parasites in DF-1 cells, staining intensified in trophozoites, immature schizonts and mature schizonts. Antibody inhibition of EtPDIL function reduced the ability of *E. tenella* to invade DF-1 cells. These results suggested that EtPDIL might be involved in sporulation in external environments and in host cell adhesion, invasion and development of *E. tenella*.

## Introduction

Avian coccidiosis is a widespread and economically significant poultry disease caused by several *Eimeria* species. This disease occurs worldwide because parasite transmission is favored by high-density housing of large numbers of susceptible birds and it costs the poultry industry millions of dollars. Although the global economic loss of the poultry industry from coccidiosis is unclear, it is estimated to be more than $3 billion per annum from production loss combined with the cost of prevention and treatment [1], [2]. Current conventional disease control strategies mainly rely on anticoccidial drugs, live wild type vaccines and live attenuated vaccines. Disadvantages of these measures include the emergence of drug resistance, consumer attention to food safety, high production expenses and danger of potential coccidiosis. The development of subunit or recombinant vaccines is also limited. Therefore, novel control approaches are urgently need to effectively control coccidiosis [3]–[5].

*Eimeria* belongs to the phylum Apicomplexa, which contains obligate intracellar parasites including medical and veterinary pathogens such as *Toxoplasma*, *Plasmodium*, *Cryptosporidium*, *Sarcocystis* and *Neospora*. These protozoan parasites are characterized by an unusual organelle complex at the apical end [6]. The life cycle of *Eimeria* is complex and consists three phases: sporogony, schizogony and gametogony. During schizogony and gametogony, which occur within the host, host cells are functionally impaired and eventually destroyed. The extent of destruction depends on the number of infective oocysts ingested, which in turn depends upon the extent of successful sporulation [1]. Thus, the sporogony phase from unsporulated oocysts to sporulated oocysts, which occurs in the external environment, is important. Unsporulated oocysts shed in chicken feces are not infectious. Once shed, oocysts undergo sporulation in the environment. This step requires warmth, oxygen, and moisture as meiotic and mitotic nuclear division produce a sporulated oocyst. The sporulated oocyst contains four sporocysts, each of which contains two infectious sporozoites [2], [7]. The developmental stages of *Eimeria* have different morphological characteristics and habitats, therefore the different life cycle stages are likely have different gene expression profiles [7]–[10]. Differentially expressed genes of sporulated oocysts might be involved in *Eimeria* sporulation and invasion. *Eimeria tenella* is an important species causing avian coccidiosis and is frequently used as a model to study *Eimeria* species. The *E. tenella* genome has been sequenced (http://www.genedb.org/Homepage/Etenella). Our previous report analyzed differentially expressed genes of *E. tenella* sporulated oocysts using suppression subtractive hybridization and cDNA microarrays and identified expressed sequence tags (ESTs) with differential expression. BLAST searches showed that a protein encoded by ESTsh009 (Genbank number: ES346888) was highly homologous to a protein disulfide isomerase (PDI)-like protein of *Plasmodium cynomolgi* and other species [9].

PDI and PDI-like proteins are members of the thioredoxin superfamily. They contain thioredoxin-like domains and catalyze the physiological oxidation, reduction and isomerization of disulfide bonds of proteins in prokaryotic and eukaryotic cells. Therefore, these proteins are involved in many aspects of cell function and development [10]. PDI and PDI-like proteins are found in plants, pathogens and humans [11]–[13]. PDI homologs also have been described in several protozoan parasites such as *Cryptosporidium parvum* [14], *Theileria parva* [15], *Plasmodium* [16], [17], *Toxoplasma gondii* [18], *Neospora caninum* [19], *Leishmania* [20], [21], *Giardia lamblia* [22] and *Trypanosoma brucei* [23], but not in *Eimeria*. The typical structure of PDI proteins is four domains (a, b, b’, a’) and a C-terminal endoplasmic reticulum (ER) resident signal peptide (KDEL or KDEL-like). Two domains (a and a’) have two thioredoxin-like motifs (CXXC) that are active sites. The two remaining domains (b and b’) interact with substrates [10] and are not active sites. The PDI gene family encodes proteins that vary in size, expression, localization, and enzymatic function [24]. Although most of PDIs described to date conform to this structural model, an increasing number of proteins in the PDI family do not follow this model. Some PDI-like proteins contain one or more thioredoxin-like active domains instead of two active domains; some have no C-terminal ER-retention signal. These atypical PDI-like proteins may have partial or no PDI activity, but can have other functions [24]–[27].

In this study, we used rapid amplification of cDNA ends (RACE) to clone a full-length cDNA sequence based on the EST sequence described above. We identified the EtPDIL gene encoding a novel, atypical PDI-like protein of *E. tenella*. EtPDIL expression was examined at different developmental stages. The involvement of EtPDIL in parasitic invasion and development was investigated through inhibition tests and immunofluorescence.

## Materials and Methods

### Parasites and *E. tenella in vitro* culture

*E. tenella* was provided by the Shanghai Veterinary Research Institute, Chinese Academy of Agricultural Sciences and maintained and propagated by passage through coccidia-free two-week-old chickens as previously described [28]. Unsporulated oocysts and sporulated oocysts were obtained and purified using standard procedures. Sporozoites were recovered from cleaned sporulated oocysts by *in vitro* excystation and purified. Second generation merozoites were collected and purified from the caecal mucosa of chickens at 112 h post inoculation [29].

The chicken embryo fibroblast cell line DF-1, derived from East Lansing Line (ELL-0) chicken embryos, was used for infection, inhibition assays and immunofluorescence experiments [30], [31]. Cells were infected at a ratio of one sporozoite per cell in complete medium (DMEM, Invitrogen, USA) containing 10% fetal calf serum (FCS) at 41°C. Cells were washed 2 h post infection and fresh medium was added.

### Cloning of EtPDI-like protein cDNA

The 773-base pair (bp) ESTsh009 (GenBank number: ES346888.1) sequence for a PDI-like protein, which is differentially expressed in *E. tenella* sporulated oocysts, was obtained using suppression subtractive hybridization and cDNA microarrays [9]. BLAST searches showed that the encoded protein had significant identity to putative PDI-like proteins of *P. cynomolgi*, *Plasmodium vivax and Eimeria mitis*. Full-length 5'- and 3'-ends of the cDNA for the gene were obtained by RACE using GeneRacer kits (Invitrogen, USA) according to manual instructions. Primers (GR5P, GR5N, GR3P and GR3N) supplied with the kit and gene-specific primers (GS5P, GS5N, GS3P and GS3N) in Table 1 were used. Gene-specific primers were designed for 5'- and 3'-ends according to the EST sequence.

**Table 1:** Primer sequence and name.

Name	Sequences (5'→3')
GR5P (GeneRace 5' Primer)	5'-CGACTGGAGCACGAGGACACTGA-3'
GR5N (GeneRace 5' Nested Primer)	5'-GGACACTGACATGGACTGAAGGAGTA-3'
GR3P (GeneRace 3' Primer)	5'-GCTGTCAACGATACGCTACGTAACG-3'
GR3N (GeneRace3' Nested Primer)	5'-CGCTACGTAACGGCATGACAGTG-3'
GS5P (Gene-specific 5' Primer)	5'-TCAGATGGGACTGGAGAAACACGAA-3'
GS5N (Gene-specific 5' Nested Primer)	5'-CTTGTGCGTCGTGAAGGCTAAGT -3'
GS3P (Gene-specific 3' Primer)	5'-ACACCACGTTGCCATTTGAGTCCTT -3'
GS3N (Gene-specific 3' Nested Primer)	5'-CCTCCGGGAACAGAATTAGGTCCAT -3'
RTS (qPCR EtPDIL Sense primer)	5'- GCGGACAAGGACGAAAGG -3'
RTA (qPCR EtPDIL Antisense primer)	5'- TCAGAGCCAACAACTACCAAG -3'
18S (qPCR 18s rRNA Sense primer)	5'-TGTAGTGGAGTCTTGGTGATTC-3'
18A (qPCR 18s rRNA Antisense primer)	5'-CCTGCTGCCTTCCTTAGATG-3'
PD1(Forward primer)	5'-GCGGATCCTTTCACTCACATTTTCTCAAGATG -3'
PD2(Reverse primer)	5'- GCGGAATTGGGCGGGTCCCTCTTAATGAACAAT -3'

All amplified fragments were gel purified (Qiagen, USA) and cloned into the pGEM-T-easy vector (Promega, USA) and transformed into *Escherichia coli* JM109 competent cells. Three positive colonies were sequenced for each 3'- and 5'- product purified from gels. The sequences of the 5'- and 3-ends of the cDNAs were compared to the original EST sequence using DNAstar software (Promega, USA). The full-length cDNA sequence was obtained and submitted to NCBI GenBank (accession number: EF552214.1).

### Sequence analysis of EtPDIL and multiple sequence alignment

The full-length cDNA sequence of the putative EtPDIL gene was analyzed using the BLAST programs at the National Center for Biotechnology Information (http://www.ncbi.nlm.nih.gov/BLAST/) and the genome sequence of *E. tenella* (http://www.genedb.org/Homepage/Etenella). The deduced amino acid sequence, molecular mass and theoretical isoelectric point were obtained using translate tool software at the ExPASy server of the Swiss Institute of Bioinformatics (http://www.expasy.org/tools/protparam.html). Signal peptides, transmembrane regions and protein motifs were predicted using SignalP (http://www.cbs.dtu.dk/services/SignalP/), TMHMM (http://www.cbs.dtu.dk/services/TMHMM-2.0/), and Motifscan (http://hits.isb-sib.ch/cgi-bin/motif_scan). Multiple sequence alignment used the program Clustal W (http://www.ebi.ac.uk/Tools/msa/clustalw2/).

### Real-time quantitative PCR of EtPDIL gene transcripts

Expression profiles of EtPDIL in *E. tenella* unsporulated oocysts, sporulated oocysts, sporozoites and second-generation merozoites were determined using real-time quantitative PCR (qPCR) on a MyiQ Two-Color Real-Time Quantitative PCR Detection System (Bio-Rad, USA) using the SYBR1 green I dye method. Total RNA was isolated with TRIzol reagent (Invitrogen, USA) from four *E. tenella* developmental stages and treated with DNase I (Invitrogen, USA). First-strand cDNA templates were generated from 2 µg total RNA by SuperScript II reverse transcriptase (Invitrogen, USA) using random primers. An *E. tenella* 18S ribosomal RNA gene fragment was used as a control. Primers for the EtPDIL gene (RTS, RTA) and 18S ribosomal RNA gene (18S, 18A) were designed manually using the Beacon Designer program (www.premierbiosoft.com). Reactions were carried out in triplicate and each experiment was performed twice. Primers for qPCR are in Table 1.

### Expression and purification of recombinant EtPDIL protein

The EtPDIL open reading frame (ORF) was amplified by RT-PCR to obtain the full-length cDNA sequence. Total RNA of sporulated oocysts were extracted and first-strand cDNA template was generated by SuperScript II reverse transcriptase (Invitrogen, USA) using oligo(dT) as primer. Sequence-specific primers were designed to contain sites for *Bam* HI in the forward primer (PD1) and *Eco*R I in the reverse primer (PD2) (Table 1). Amplified fragments were cloned into the pGEM-T-easy vector (Promega, USA) and sequenced. Recombinant plasmids and expression vectors pGEX-4T-2 (Novagen, USA) were digested with *Bam* HI and *Eco*RI. Fragments were cut from gels and purified using a QIAquick Gel Extraction kit (Qiagen, USA), and ligated with T4 DNA ligase (Promega, USA), then transformed into competent *E. coli* BL21(DE3) (Tiangen, China).

Recombinant protein expression from *E. coli* clones identified by sequencing was induced using 1 mM isopropylthio-α-D-galactoside (IPTG) (Sigma-Aldrich, USA) at OD_600_ = 0.6. Induced bacterial cells were incubated for 6 h and harvested by centrifugation. Cell lysates were prepared using lysozyme (10 µg/ml) (Sigma-Aldrich, USA) and sonication and analyzed by 12% SDS-PAGE to confirm the distribution of expressed recombinant protein. Recombinant EtPDIL (rEtPDIL) protein was purified from lysate supernatants using GST Bind Resin (Merck, Germany). Yield of affinity-purified protein was estimated using a Biophotometer (Eppendorf, Germany). Purified rEtPDIL was visualized by 12% SDS-PAGE after staining with Coomassie brilliant blue. Purified recombinant protein was stored in aliquots at −20°C.

### Polyclonal sera against recombinant EtPDIL protein

rEtPDIL protein was used to immunize a 2-month-old male rabbit by intraperitoneal injection of 200 µg purified recombinant protein emulsified in Freund's complete adjuvant (Sigma-Aldrich, USA). The rabbit was boosted 2 weeks later with purified recombinant proteins emulsified in Freund's incomplete adjuvant (Sigma-Aldrich, USA) under the same conditions and three additional times at intervals of 2 weeks. One week after final immunization, antiserum against rEtPDIL was collected. All polyclonal antibodies used for inhibition of infection experiments were purified from rabbit sera using Protein A+G agarose (Beyotime, CN).

### SDS-PAGE and Western blots

Protein lysates from *E. tenella* unsporulated oocysts, sporulated oocysts, sporozoites and second-generation merozoites were prepared in cell lysis buffer for Western and IP (Beyotime, CN). Protein concentration was determined by a BCA protein assay kit (Beyotime, CN). Protein lysates and rEtPDIL protein were resolved by 12% SDS-PAGE and transferred to polyvinylidene difluoride membranes (Millipore, USA). Western blots were performed according to standard procedures [32]. Primary antibodies were rabbit sera against sporulated oocysts or rEtPDIL protein at 1∶100. Anti-GST monoclonal antibody or mouse monoclonal anti-α-tubulin (1∶2000) (Sigma, USA) or naive rabbit serum (1∶100) were used as negative controls. Horseradish peroxidase-conjugated goat anti-rabbit IgG or goat anti-mouse IgG (1∶2000, Sigma, USA), IRDye 800CW goat anti-rabbit IgG (LI-COR, Biosciences, USA 1∶10000) or IRDye 680RD donkey anti-mouse IgG (LI-COR, Biosciences, USA 1∶10000) were second antibodies. Peroxidase activity was determined with diaminobenzidine (Sigma-Aldrich, USA) or membranes were scanned with an Odyssey Infrared Imaging System (LI-COR, Biosciences, USA).

### Immunofluorescence of EtPDIL during development

Sporozoites were purified and incubated for 2 h at 41°C in PBS or complete medium (Invitrogen, USA) and air-dried on a glass slide before fixation [30], [32]. Sporozoites incubated in complete medium were inoculated into DF-1 cells. At various times post inoculation, DF-1 cells were collected and washed. Sporozoites and cells were fixed with 2% paraformaldehyde in PBS and permeabilized with 1% TritonX-100 in PBS for 15 min at room temperature, then blocked with PBS containing 2% (w/v) bovine serum albumin for 20 min. Rabbit antiserum against rEtPDIL protein (1∶100) was added and incubated for 1 h at 37°C. After washing three times in PBS, goat anti-rabbit IgG fluorescein isothiocyanate-conjugated antibody (1∶500; Sigma-Aldrich, USA) was added for 1 h at 37°C. Cell nuclei were labeled with 10 µg/mL DAPI (40, 6-diamidino-2-phenylindole, Beyotime, CN) for 10 min. At each step, sporozoites and cells were washed three times in PBS, all dilutions and washes used 0.05% Tween 20-PBS. Slides were treated with 60 µL Fluoromount Aqueous Mounting Medium (Sigma-Aldrich, USA) and observed with a florescence microscope (Nikon, Japan).

### Invasion inhibition assay

Invasion inhibition assays were based on invasion of DF-1 cells by *E. tenella* sporozoites [30], [33]. Freshly isolated sporozoites were irreversibly labeled with carboxyfluorescein diacetate succinimidyl ester (CFSE-Molecular Probes, Beyotime, CN) according to the manufacturer's instruction. Sporozoites (1×10^7^) were diluted in 2 mL sterile PBS with 2 µL 1 mM CFSE for a final concentration of 2 mM. After 30 min, sporozoites were washed twice with DMEM (Invitrogen, USA) supplemented with 2.5% FCS (Invitrogen, USA). Labeled sporozoites were preincubated at 37°C with 50 µg/mL, 100 µg/mL, 200 µg/mL or 300 µg/mL purified anti-rEtPDIL IgG for 2 h in 1 mL complete medium, then washed twice with DMEM. Controls were the same volume of purified IgG from naive rabbit sera, or untreated sporozoites. Sporozoites (1×10^5^/well) were used to infect 1×10^5^ DF-1 cells in 24-well plates (Corning, USA).

After 16 h at 41°C, infected cells were trypsinized, washed, and fixed in 2% paraformaldehyde in PBS for 10 min. Cells were washed 3 times with PBS and analyzed by flow cytometry using a Cytomics FC 500 (Beckman Coulter, USA). Controls were noninfected DF-1 cells. Infected cells, noninfected cells, and free sporozoites were gated using RXP software for counting infected (labeled sporozoites) and uninfected (fluorescence-free) cells. All assays were performed in triplicate. Percentages of infected cells in the presence or absence of inhibitory antibody were used to calculate inhibition rates as previously described [30], [33].

### Statistical analysis

Statistical analysis used the SPSS statistical package (SPSS for Windows 16, SPSS Inc., Chicago, IL, USA). All data, including real-time qPCR and invasion inhibition assay results, were analyzed. Differences among groups were tested by one-way analysis of variance (ANOVA) Duncan test. P<0.05 was considered significant and P<0.01 highly significant.

### Ethics Statement

Coccidia-free Chickens and male rabbit were used in this study. The protocol was approved by the Animal Care and Use committee of the Shanghai Veterinary Research Institute, Chinese Academy of Agricultural Sciences. The animals were provided with water and food *ad libitum*. At the end of the experiments, the animals were euthanized in strict accordance with the international standards for animal welfare.

## Results

### Cloning and sequence analysis of EtPDIL full-length cDNA

**Figure 1:**
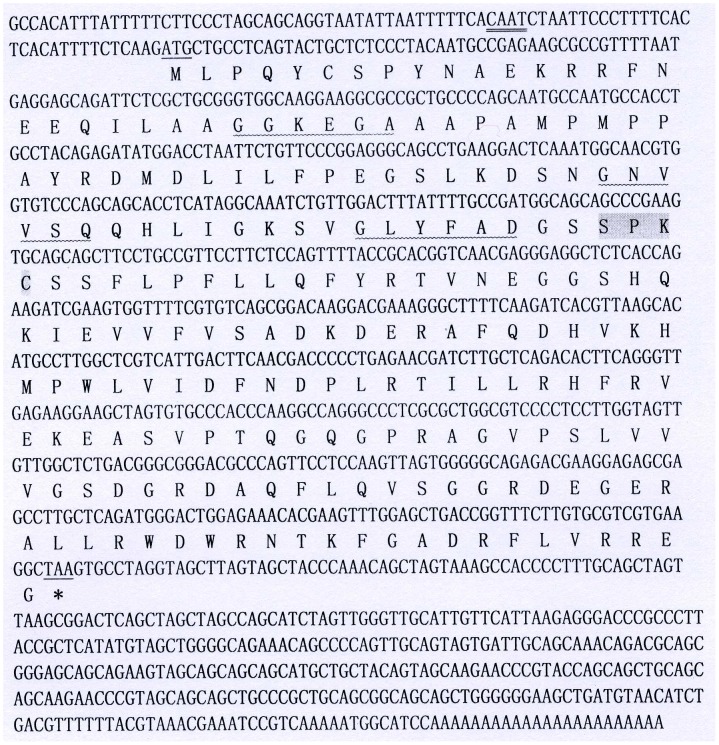
Full cDNA and deduced amino acid sequence of EtPDIL. Underlined, start and stop codons; double underlined, CAAT box in the 5′-UTR; gray, non-classical thioredoxin-like motif (SXXC); wavy underlined, N-myristoylation site.

ESTsh009, with high homology to PDI-like genes, was 773 bp without the cDNA 3'- or 5'-ends. Using 5'- and 3'-RACE primers, the unknown ends were cloned. Overlapping the RACE fragments and the original EST sequence yielded the full-length cDNA sequence by RT-PCR. The 1129 nucleotides of the full-length cDNA were sequenced. The full-length cDNA included a 5'-untranslated region (UTR) of 82 bp with a CAAT box and no TATA box, a 3'-UTR of 396 bp with a poly(A) tail and an ORF of 651 bp (positions 83–733) encoding 216 amino acids with a predicted molecular mass of 24.15 kDa and a theoretical isoelectric point of 6.97 (Fig. 1). The deduced amino acid sequence had no predicted signal peptide, transmembrane region or C-terminal ER resident signal peptide, but had three N-myristoylation sites, two protein kinase C phosphorylation sites and two casein kinase phosphorylation sites.

Motifscan predicted a single thioredoxin domain between amino acids 26-204, belonging to the thioredoxin-like superfamily. Instead of two classical redox-active CXXC motifs of PDI within the thioredoxin domains the sequence had a non-classical thioredoxin-like motif (SXXC). A BLAST search of the *E. tenella* genome database (http://www.genedb.org/Homepage/Etenella) found that the ORF sequence had 100% (651/651) identity with ETH_00030030 on supercontig_14, encoding a conserved hypothetical protein with a thioredoxin-like fold.

**Figure 2:**
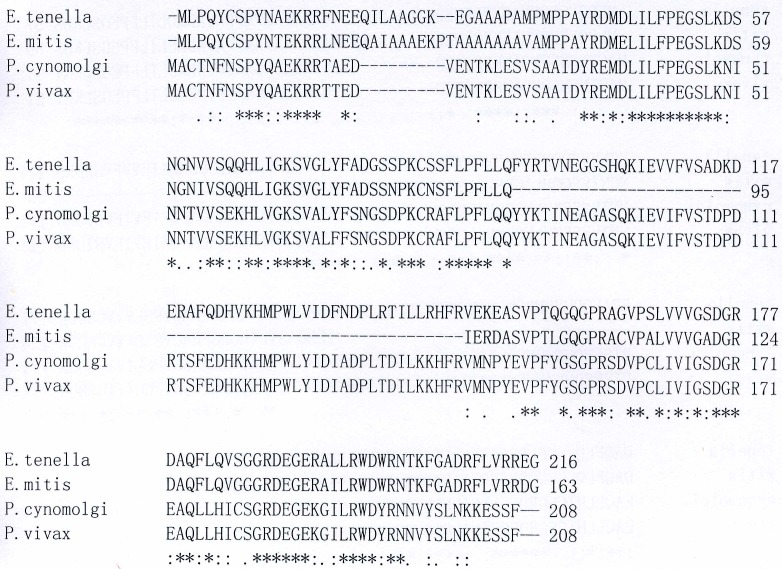
Multiple alignment analysis of EtPDIL of *Eimera tenella* with PDIL from other apicomplexan parasites. Shown are sequences from *Plasmodium cynomolgi* (XP_004221713), *Plasmodium vivax* (XP_001614725), *Eimeria mitis* (CDJ34317.1). Deduced protein sequences were used in the Clustal W sequence alignment program. Asterisks, identical amino acids.

Database searches revealed that the predicted amino acid sequence from the EtPDI-like gene was highly homologous to other apicomplexan PDI-like protein including PDI-like proteins from *P. cynomolgi* (Genbank number: XP_004221713, 56% identity and 70% similarity), *P. vivax* (Genbank number: XP_001614725, 55% identity and 70% similarity) and *E. mitis* (Genbank number: CDJ34317.1, 59% identity and 66% similarity) (Fig. 2). The gene was designated EtPDIL (GenBank accession no. EF552214).

### EtPDIL transcripts at different *E. tenella* developmental stages

**Figure 3:**
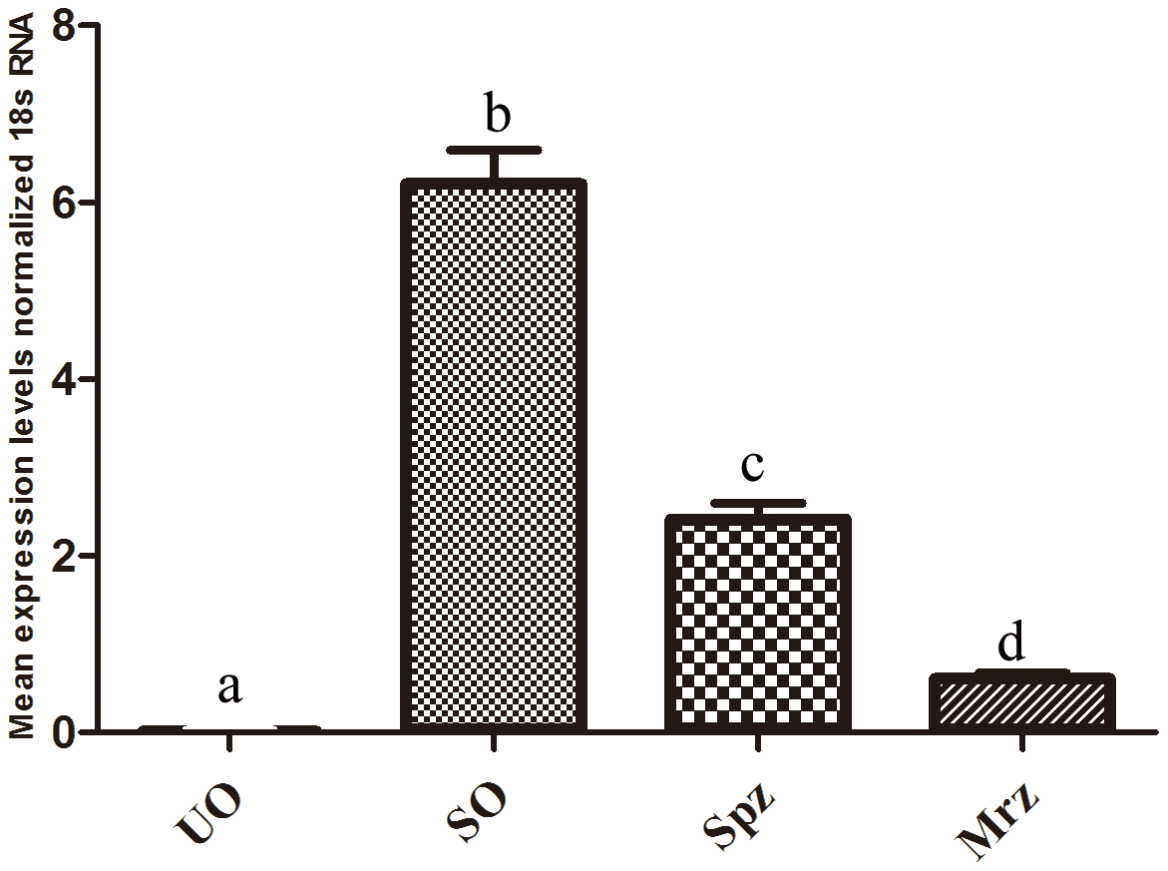
Quantitative real-time RT-PCR of EtPDIL expression in *E. tenella* developmental stages. UO, unsporulated oocysts; SO, sporulated oocysts; Spz, sporozoites; Mrz, merozoites. Bars not sharing the same letters were significantly different (P<0.05).

EtPDIL transcripts were detected in unsporulated oocysts, sporulated oocysts, sporozoites, and second generation merozoites by qPCR. The transcript levels of EtPDIL were higher in sporulated oocysts than in the unsporulated oocysts, sporozoites or second generation merozoite. Transcript levels were lowest in the unsporulated oocysts (Fig. 3).

### Expression and identification of recombinant EtPDIL

**Figure 4:**
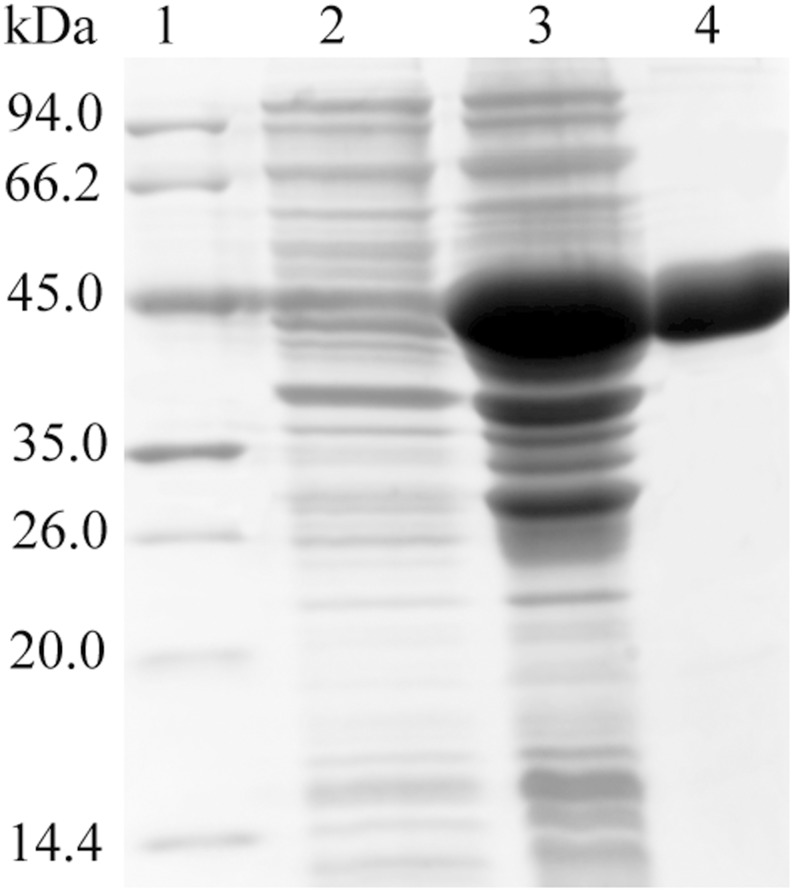
Expression of recombinant EtPDIL in *Escherichia coli* by SDS-PAGE. Lane 1, protein marker; lane 2, IPTG-induced recombinant EtPDIL protein at 0 h; lane 3, IPTG-induced recombinant EtPDIL protein at 6 h; lane 4, purified recombinant EtPDIL.

**Figure 5:**
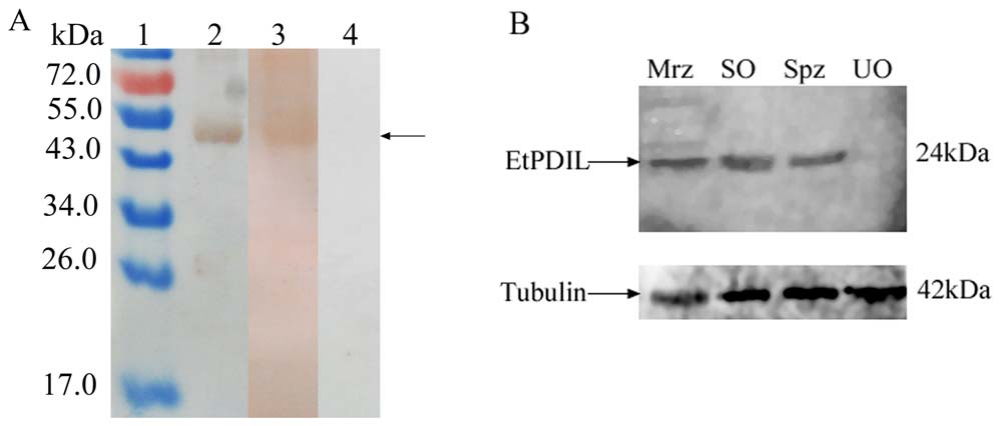
Western blots. (A) Purified recombinant EtPDIL (diaminobenzidine as substrate). Rabbit sera against sporulated oocysts of *E. tenella* or anti-GST monoclonal antibody was used as primary antibody. Lane 1, protein marker. Lane 2, anti-GST monoclonal. Lane 3, antisporulated-oocysts serum. Lane 4, naive rabbit serum. (B) Protein lysates from four different life cycle stages of *E. tenella.* Rabbit sera against rEtPDIL or mouse monoclonal anti-α-tubulin was used as primary antibody. Spz, sporozoites; SO, sporulated oocysts; UO, unsporulated oocysts; Mrz, merozoites.

rEtPDIL was expressed in *E. coli* as a GST-tagged fusion protein. The recombinant protein was mainly found in lysate supernatants. After purification by affinity chromatography using glutathione sepharose media, a protein band of approximately 50 kDa was detected (Fig. 4). Because 26 kDa of the fusion protein was from the vector and the predicted molecular mass of EtPDIL protein was about 24 kDa. Western blots showed that the purified protein was recognized by rabbit serum against sporulated oocysts or anti-GST monoclonal antibody. Naive rabbit sera failed to detect any protein of the expected size of rEtPDIL (Fig. 5A). These results indicated that rEtPDIL was recognized specifically by rabbit sera against sporulated oocysts and anti-GST monoclonal antibody.

### EtPDIL protein at different *E. tenella* developmental stages

The presence of EtPDIL in unsporulated oocysts, sporulated oocysts, sporozoites and second generation merozoites was determined by immunoblotting using rabbit antiserum against rEtPDIL. Mouse anti-α-tubulin monoclonal antibody was used as control. In Western blots, anti-rEtPDIL reacted with parasite lysates from *E. tenella* sporulated oocysts, sporozoites and second-generation merozoites showing a band of approximately 24 kDa, close to the predicted EtPDIL size of 24.1 kDa. No protein was detected in unsporulated oocysts (Fig. 5B).

### Immunofluorescence of EtPDIL in *E. tenella*-infected DF-1 cells

**Figure 6:**
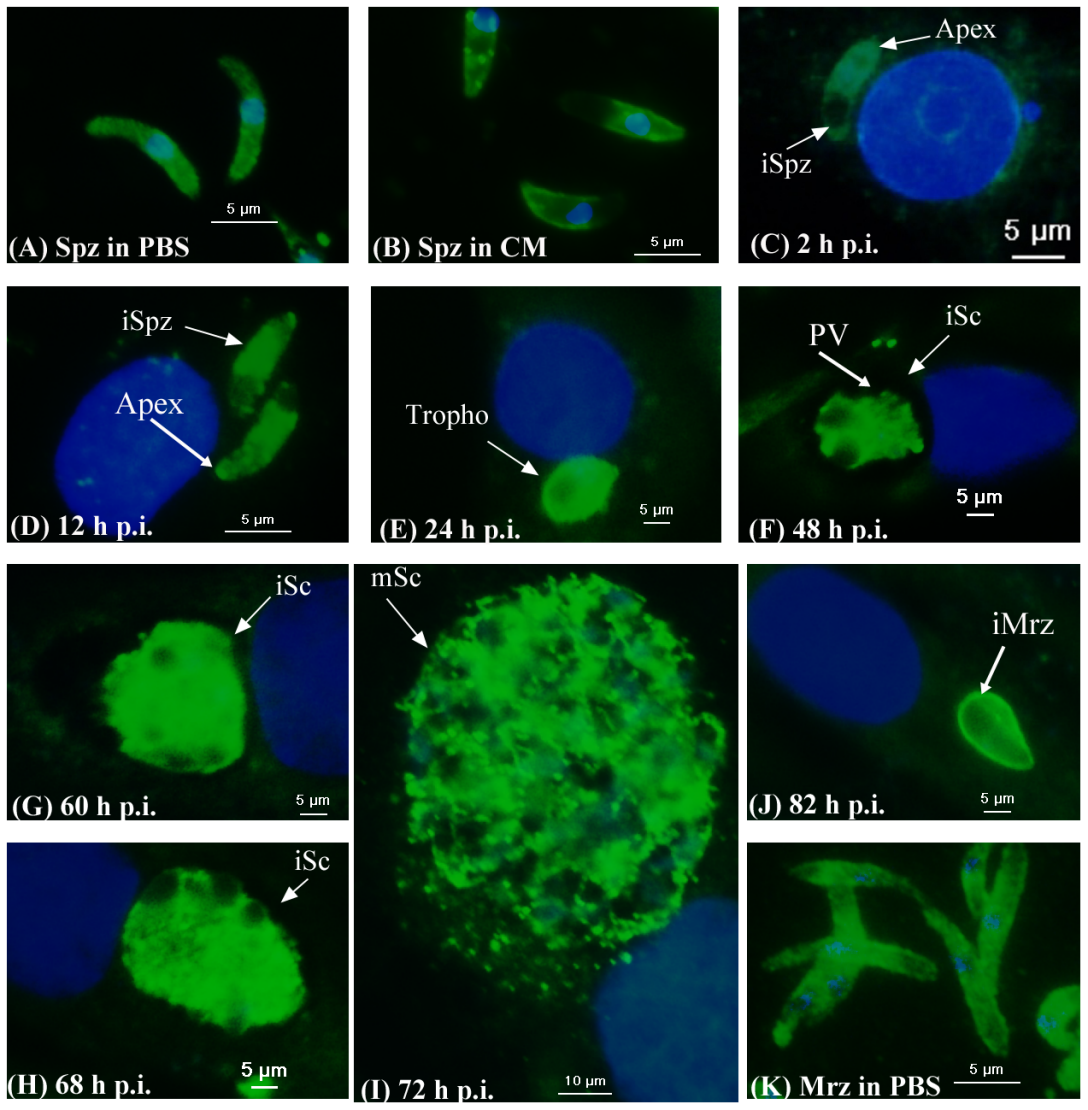
Localization of EtPDIL in different stages of *E. tenella* by indirect immuofluorescence using rEtPDIL antibody. (A) Sporozoites (Spz) in PBS; (B) Spz in complete medium. Infected DF-1 cells were collected at indicated hours post infection (p.i.). (C) 2 h p.i., intracellular sporozoites (iSpz); (D) 12 h p.i., intracellular sporozoites (iSpz); (E) 24 h p.i., trophozoites (Tropho); (F) 48 h p.i., immature schizonts (iSc); (G) 60 h p.i., immature schizonts (iSc); (H) 68 h p.i., immature schizonts (iSc); (I) 72 h p.i., mature schizonts (mSc); (J) 85 h p.i., intracellular merozoites (iMrz); (K) Merozoites (Mrz) in PBS.

Using antibody against rEtPDIL, EtPDIL was localized in sporozoites, merozoites and during first schizogony *in vitro* by immunofluorescence. EtPDIL had a disperse distribution in the cytoplasm of whole sporozoites after incubation in PBS (Fig. 6A). In sporozoites incubated in complete medium, EtPDIL was mainly concentrated at the anterior of sporozoites and on the parasite surface (Fig. 6B). After sporozoites were added to DF-1 cells for 2 h or 12 h, EtPDIL was located in the cytoplasm and on the surface of intracellular sporozoites with intense specific staining, but not in the posterior refractile body (Fig. 6C and 6D). When sporozoites developed into trophozoites in DF-1 cells, specific staining was more intense (Fig. 6E). At later times post infection, staining showed a uniform distribution in immature and mature schizonts. Staining was more intense in immature schizonts and mature schizonts (Fig. 6F, 6G and 6H). EtPDIL was not detected in parasitophorous vacuoles (PV) or the parasitophorous vacuole membrane (PVM) (Fig. 6F and 6G). After first-generation merozoites from mature schizonts reinvaded DF-1 cells, labeling became stronger and was mainly located on the parasite surface (Fig. 6J). In addition, EtPDIL showed a homogenous distribution in the cytoplasm of second-generation merozoites purified from infected chicken ceca (Fig. 6K).

### Inhibition of *E. tenella* invasion by antibodies against recombinant EtPDIL

**Figure 7:**
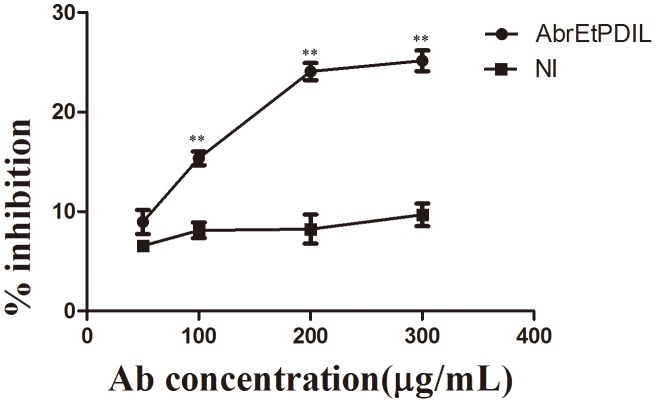
Inhibition of sporozoite invasion *in vitro* by antibody against rEtPDIL. Anti- rEtPDIL, rabbit antiserum against recombinant EtPDIL protein; NA, naive rabbit serum. All assays were performed in triplicate. ** P<0.01 for differences between treatment with antibody against rEtPDIL and naïve rabbit serum at the same IgG concentration.

To study EtPDIL protein in *E. tenella* sporozoite invasion of DF-1 cells, invasion inhibition assays were performed by blocking sporozoites by preincubation with rEtPDIL antibody before DF-1 cell infection. Pretreatment with antibody significantly decreased the invasion capacity of the sporozoites. Compared with the same dose of naive rabbit sera IgG used as a negative control, pretreatment with 50 µg/mL anti-EtPDIL IgG did not significantly affect the invasion capacity of sporozoites (P>0.05). However, pretreatment with 100 µg/mL, 200 µg/mL or 300 µg/mL anti-EtPDIL IgG significantly decreased invasion (P<0.01). Inhibition was as high as 25% at 300µg/mL anti-EtPDIL IgG. The observed inhibition was dose dependent with an inhibition plateau of 23–25% at 300 µg/mL. Naive rabbit sera IgG did not have a significant effect on invasion (Fig. 7).

## Discussion

In this work, the full-length cDNA of a new PDI-like protein, EtPDIL, was cloned from *E. tenella* was based on a single EST. The gene encoding EtPDIL was 1129 bp with a 651 bp ORF encoding 216 amino acids. The sequence included a 5'- UTR of 82 bp, a 3'-UTR of 396 bp with a poly(A) tail and no canonical polyadenylation signal sequence AATAA. The deduced amino acid sequence had no signal peptide, transmembrane regions or C-terminal ER-retention signal. BLAST analysis revealed that EtPDIL was highly homologous to other apicomplexan PDI-like proteins from *P. cynomolgi*, *P. vivax,* and *E. mitis*. Motifscan indicated that EtPDIL protein has a single predicted thioredoxin-like domain with a non-classical SXXC motif. Classical PDI proteins have two thioredoxin–like motifs (CXXC) that contain an independent active site and an ER retention signal (KDEL or KDEL-like) at the C-terminus. Each active site contains two cysteines that mediate PDI activity [13], [34]. A number of proteins in the PDI family do not follow this model. Some PDI family members contain one or more thioredoxin–like motifs [10], [12], [24]. Genes encoding PDIs and PDI-like proteins with a single thioredoxin-like domain have been reported in many species, including plants [11], bacteria [35], yeast [36], fungi [37], protozoans [21] and humans [24]. Here, we showed the apicomplexan parasite *E. tenella* possesses a PDI-like protein with a single thioredoxin-like domain. EtPDIL lacks the two typical redox-active CXXC motifs of PDI and contains only an SXXC sequence within a thioredoxin-like domain. However, the CXXC motif is not invariant in PDIs [38]. Several non-classical thioredoxin-like motifs have been reported, such as CXXS, SXXS and SXXC [25], [39], [40]. Eug1p, a yeast PDI, is a typical example of a protein with thioredoxin-like domains with non-classical CXXS motifs. The presence of serine instead of the second cysteine of CXXS decreases the oxidative refolding and isomerase activity [39]. Human PDILT (a divergent testis-specific PDI) has two non-classical SXXC and SXXS motifs. PDILT is not able oxidize or reduce disulfide bonds, although some evidence suggests that PDILT forms in disulfide-bonded complexes *in vitro* [25]. EtPDIL only contains a single non-classical SXXC motif, the disulfide isomerase activities of recombinant EtPDIL were measured by re-activation of scrambled RNase (sRNase) according to the previous reported procedures [41], [42], but the results showed that the recombinant EtPDIL has no enzymatic activities. Our results were in accordance with the previous reports, the first (N-terminal) cysteine in either active site (CXXC) is essential for catalysis of oxidation and rearrangement during the refolding of reduced bovine pancreatic ribonuclease A (RNase). Mutant active sites with the sequence SXXC show no detectable activity for disulfide formation or rearrangement [43]. These results suggest that both cysteines in the CXXC motif of PDI are required for PDI-catalyzed oxidase activity. In fact, evidence suggests that although eukaryotes have multiple PDI homologs, only subsets catalyze disulfide bond formation [44].

Transcriptional and protein expression of EtPDIL was determined in unsporulated oocysts, sporulated oocysts, sporozoites and second-generation merozoites. Results from qPCR and western blots showed that EtPDIL mRNA and protein levels were highest in sporulated oocysts and lowest in unsporulated oocysts; protein expression was nearly undetectable in unsporulated oocysts. These results showed that EtPDIL was expressed at a distinct phase of the parasite life cycle, consistent with previous reports [9]. The EtPDIL EST was a differentially expressed gene obtained from sporulated oocysts using suppression subtractive hybridization and cDNA microarrays; qPCR showed that the EST was highly expressed in sporulated oocysts [9]. In other protozoan parasites, stage-specific expression PDIs have been observed in different developmental stages. For example, in *T. brucei*, TbPDI-1 and TbPDI-2 expression is developmentally regulated at the mRNA and protein level and is restricted to the bloodstream forms of the parasite with no expression in the procyclic forms that are the major proliferative stage in the tsetse fly vector [23]. EtPDIL expression was also not detected in unsporulated oocysts. The same expression pattern was found in *Plasmodium falciparum*: PfPDI-9 and-14 are absent from the sporozoite stage but expressed in schizonts and gametes, and PfPDI-8 and 11 are expressed during all stages of parasite development [16], [40]. In *N. caninum*, expression of NcPDI is downregulated during tachyzoite-to-bradyzoite stage conversion, suggesting that the protein might be involved in tachyzoite-host cell interaction [19]. Previous reports also indicated that the expression of PDI-2 in *G. lamblia* trophozoites is higher than in cysts; this protein is involved in encystment [45], [46]. EtPDIL mRNA and protein levels was the highest in in sporulated oocysts, so the protein might be involved in sporulation of *E. tenella* in the environment.

By indirect immunofluorescence with an antibody against rEtPDIL, we showed that the protein was located in the cytoplasm of *E. tenella* sporozoites and merozoites. However, EtPDIL was on the surface and at the apical end of sporozoites after incubation in complete medium. EtPDIL was also on the surface of the first merozoites released from mature schizonts after invading DF-1 cells. Most PDIs are localized mainly in ER and are involved in cell signaling and homeostasis [47]. Some PDIs and PDI-like proteins are in other intracellular compartments [48] such as the plasma membrane [49] or organelles [50] or are secreted [19]. For instance, in *N. caninum* and *T. gondii*, PDIs are expressed on the surface of tachyzoites and involved in tachyzoite-host cell interaction and functionally influence the adhesive and invasive capacities of tachyzoites [18,19 51]. *G. lamblia* PDI is expressed on the surface and is involved in protecting the protozoan from environmental oxidative stress, which is considered to be a survival mechanism [22]. EtPDIL was found on the parasite surface. Although the protein had no transmembrane region, it had three N-myristoylation site. Myristoylation is crucial for membrane targeting and signal transduction in the plant response to environmental stress [52]. In *N. caninum*, NcPDI is expressed on the surface of tachyzoites, but has no membrane-spanning domain and can be released from live tachyzoites using sodium carbonate [19]. EtPDIL has no C-terminal ER-retention signal, which is found in PDIs or PDI-like proteins of other species, but immunofluorescence showed a disperse distribution of EtPDIL in the cytoplasm of sporozoites and second-generation merozoites. However, some parasites PDIs, for example in *Leishmania donovan* and *Clonorchis sinensis*, have no C-terminal ER-retention signal but are located, at least in part, in the ER and are critical in the secretory pathway [21], [53].

Some PDIs are abundant in *Plasmodium* and *N. caninum* micronemes and are important for binding to host cells and for parasite invasion [19], [50]. Micronemes are the smallest of the apicomplexan secretory organelles that cluster at the apical end of the invading stages of all apicomplexan parasites. Micronemes contain proteins that are critical for parasite adhesion to host cells. Microneme secretion is rapidly upregulated when parasites contact host cells [54]. Whether EtPDIL protein is presented in *E. tenella* micronemes is not clear and further studies are necessary to investigate this. EtPDIL protein was not expressed in unsporulated oocysts but was mainly located on the sporozoite surface after incubation in complete medium and in merozites after invasion of DF-cells. When parasites developed in DF-1 cells, EtPDIL staining was more intense in trophozoites and mature schizonts. These results suggested that EtPDIL was involved in adhesion and invasion of parasites in the host cell or might be important role for survival of parasitic intracellular stages.

Our previous studies showed that *in vitro* invasion inhibition assays reduced *E. tenella* sporozoite invasion with specific polyclonal antibodies [30], [31], [55], [56]. Western blots showed that rabbit anti-rEtPDIL antibody recognized a single band of the expected 24 kDa in *E. tenella* sporozoites, merozoites and sporulated oocysts, but not unsporulated oocysts. So we assumed that anti-EtPDIL was specific to EtPDIL. Invasion inhibition assays using rEtPDIL antibody showed partial blocking of the invasion of sporozoites into DF-1 cells. Previous reports indicated that incubation of *N. caninum* tachyzoites with NcPDI antibodies reduced host cell adhesion and invasion [19]. These results further supported EtPDIL involvement in adhesion and invasion of *E. tenella* to host cells. Another, some PDI and PDI-like proteins have special functions in ampicomplexan parasites. For instance, TgPDI of *T. gondii* is a novel vaccine candidate against toxoplasmosis because immunization with recombinant TgPDL elicits a significant protective immune reaction [57]. In *Leishmania major*, LmPDI is a virulence factor that is important in natural pathogenicity and might be a target for new anti-Leishmania drugs [20], [58]. Thus, EtPDIL might have unknown functions.

In summary, a full-length cDNA of novel PDI-like protein, EtPDIL of *E. tenella* was cloned, expressed and characterized. Our results suggested that the protein might be important in parasite sporulation in external environments, host cell adhesion and invasion, and development in host cells. Further studies are needed to determine the exact function of EtPDIL, which might be a target for chemotherapy against *Eimeria*.
